# Shifts in methanogen community structure and function across a coastal marsh transect: effects of exotic *Spartina alterniflora* invasion

**DOI:** 10.1038/srep18777

**Published:** 2016-01-05

**Authors:** Junji Yuan, Weixin Ding, Deyan Liu, Hojeong Kang, Jian Xiang, Yongxin Lin

**Affiliations:** 1 State Key Laboratory of Soil and Sustainable Agriculture, Institute of Soil Science, Chinese Academy of Sciences, Nanjing 210008, China; 2 University of Chinese Academy of Sciences, Beijing 10049, China; 3 School of Civil and Environmental Engineering, Yonsei University, Seoul 120–749, Korea

## Abstract

Invasion of *Spartina alterniflora* in coastal areas of China increased methane (CH_4_) emissions. To elucidate the underlying mechanisms, we measured CH_4_ production potential, methanogen community structure and biogeochemical factors along a coastal wetland transect comprised of five habitat regions: open water, bare tidal flat, invasive *S. alterniflora* marsh and native *Suaeda salsa* and *Phragmites australis* marshes. CH_4_ production potential in *S. alterniflora* marsh was 10 times higher than that in other regions, and it was significantly correlated with soil organic carbon, dissolved organic carbon and trimethylamine concentrations, but was not correlated with acetate or formate concentrations. Although the diversity of methanogens was lowest in *S. alterniflora* marsh, invasion increased methanogen abundance by 3.48-fold, compared with native *S. salsa* and *P. australis* marshes due to increase of facultative *Methanosarcinaceae* rather than acetotrophic and hydrogenotrophic methanogens. Ordination analyses suggested that trimethylamine was the primary factor regulating shift in methanogen community structure. Addition of trimethylamine increased CH_4_ production rates by 1255-fold but only by 5.61- and 11.4-fold for acetate and H_2_/CO_2_, respectively. *S. alterniflora* invasion elevated concentration of non-competitive trimethylamine, and shifted methanogen community from acetotrophic to facultative methanogens, which together facilitated increased CH_4_ production potential.

Climate change and exotic plant invasions are major environmental changes that threaten the sustainability of global ecosystems^1^. Invasive plant species can alter biodiversity and stability of ecosystems worldwide^2^ and are increasingly expensive to control^3^. Furthermore, it is predicted that climate change and invasive plants can interact synergistically^4^. Climate change is expected to increase the risk of plant invasions through ecosystem disturbance and via the enhanced competitiveness of invasive species under elevated CO_2_ levels, and increased global temperatures, precipitation, and nutrient availability^1^. Meanwhile, invasive plants can participate in feedback loops that affect climate change by regulating the sequestration and stabilization of soil organic carbon (SOC) and greenhouse gas emissions^1^,^5^,^6^.

CO_2_ emissions from soils change in response to plant invasions^7^, while microbial-mediated atmospheric non-CO_2_ trace gases are also affected by invasive plants^5^,^8^. For example, plant invasions enhance soil N_2_O emissions by increasing the abundance and changing the composition of ammonia-oxidizing bacteria, thus, increasing nitrification^5^,^9^. In contrast, the response of CH_4_ emissions to plant invasions remains unclear. Cheng *et al.*^10^ and Zhang *et al.*^8^ found that the invasion of *Spartina alterniflora* to China’s coastal salt marshes significantly stimulated CH_4_ emissions compared with pristine areas containing native *Suaeda salsa* and *Phragmites australis*. The invasion of the European *P. australis* haplotype to the east coast of the United States, however, did not affect CH_4_ emissions compared with areas containing native *S. alterniflora*^11^. Differences in belowground biomass or SOC storage in invaded ecosystems are thought to cause this discrepancy in CH_4_ emissions between marshes in China and North America^10^,^11^.

Methanogenesis is the terminal step in the anaerobic decomposition of organic matter. Hence, the rate and pathway of methanogenesis reflect the community structure of methanogenic archaea because different species of methanogens exhibit different kinetics and different responses to environmental conditions^12^,^13^. For example, Liu *et al.*^14^ reported that CH_4_ production potential increased as the dissolved organic carbon (DOC) concentration gradient increased following a shift of dominant methanogens from acetotrophic *Methanosaetaceae* to hydrogenotrophic *Methanobacteriales* in freshwater wetlands. Godin *et al.*^13^ also found that CH_4_ emissions were greater in the *Methanomicrobiales*-dominated, nutrient-rich fens than in the *Methanosaetaceae*-dominated, nutrient-poor fens in Canada.

Methanogen community structure in wetlands is affected by environmental factors such as vegetation types^15^, temperature^16^, water table^17^, pH^18^ and the presence of favorable electron acceptors^19^. Hence, shifts of the dominant plant species in wetlands can directly or indirectly influence the abundance and community structure of methanogens by providing substrates through root exudates, root debris and litters^15^. This process is more severe with invasive plants as they are often more efficient at acquiring and using resources and, consequently, exhibit a higher net primary productivity and biomass than do native plants^20^. Additionally, invasive plants exhibit distinct litter chemistries compared with native species, which may have disproportionate impacts on CH_4_ production and the community structure of methanogens^21^. Therefore, information about the community structure and activity of methanogens is essential to better understand the process by which plant invasions alter environmental conditions, change the availability of substrates and ultimately affect CH_4_ emissions in invaded ecosystems.

*S. alterniflora* is a C_4_ plant native to the Atlantic and Gulf coastal marshes of North America that was intentionally introduced to China in 1979 to control coastal erosion and stabilize sediment^22^. By 2007, this species had spread by replacing native species over an area of 34,451 ha^23^, constituting 3.54% of coastal wetlands in China^24^. Previous studies have shown that *S. alterniflora* invasions considerably increase SOC storage^6^, levels of nitrogen^25^ and sulfur^26^, and the overall rates of nutrient cycling in soil^27^. All such changes can potentially affect CH_4_ production either by increasing the substrates for methanogens or by increasing sulfates and other preferred electron acceptors in the invaded salt marshes^19^,^21^. In this study, we examined the methanogen community structure and CH_4_ product potential of five coastal marsh habitats (each containing invasive *S. alterniflora*, native *S. salsa* and *P. australis*, unvegetated bare tidal flat and open water sediments) to address the following three questions: (1) does the *S. alterniflora* invasion alter methanogen community structure; (2) which factors induce changes in methanogen community structure; and (3) do shifts in methanogen community structure alter CH_4_ product potential?

## Results

### Plant aboveground biomass and soil characteristics

The aboveground biomass of *S. alterniflora* was significantly higher (1.70 kg m^−2^) than those of *P. australis* (0.79 kg m^−2^) and *S. salsa* (0.41 kg m^−2^; Table 1). SOC concentration was 1.50–9.21 times higher in the *S. alterniflora* marsh region than in any of the other four regions, while the two unvegetated regions had the lowest SOC concentrations. DOC concentration in *S. alterniflora* marsh was 1.07 g C kg^−1^, 2.36–6.19 times higher than in the other regions. Soil salinity in the coastal salt marsh varied from 3.16–16.1‰. Sulfate concentration, meanwhile, varied from 0.25–1.22 g SO_4_^2−^ kg^−1^ and was greatest in the *S. alterniflora* marsh, followed by the open water sediments, bare tidal flat, *S. salsa* marsh, and *P. australis* marsh. Trimethylamine concentration in the *S. alterniflora* marsh was 2.34–18.4 times higher than in the other regions, while acetate concentration was highest in the *P. australis* marsh. Both trimethylamine and acetate concentrations were lowest in the unvegetated regions. Mean formate concentration was highest in the *S. alterniflora* and *S. salsa* marshes, though the differences among the five regions were not significant. SOC was significantly correlated with DOC (*R*^2^ = 0.931, *P* = 0.008, *n* = 5) and trimethylamine (*R*^2^ = 0.922, *P* = 0.009, *n* = 5) but not correlated with acetate (*R*^2^ = 0.262, *P* = 0.378, *n* = 5) or formate concentration (*R*^2^ = 0.302, *P* = 0.338, *n* = 5).

### Abundance and community structure of methanogens

The abundance of methanogens as estimated by real-time PCR varied from 0.26 × 10^8^ to 3.56 × 10^8^ copies g^−1^ d.w.s (gram dry weight soil) in the five regions of the transect (Table 2). Methanogen abundance in the *S. alterniflora* marsh was approximately an order of magnitude greater than in the two unvegetated regions and between 83.9% and 105% greater than in the *P. australis* and *S. salsa* marshes. When data from the five regions were combined, the abundance of methanogens was significantly correlated with SOC, TN, DOC and trimethylamine, but not correlated with soil salinity, sulfate, acetate or formate concentrations (Table S1).

**Figure 1:**
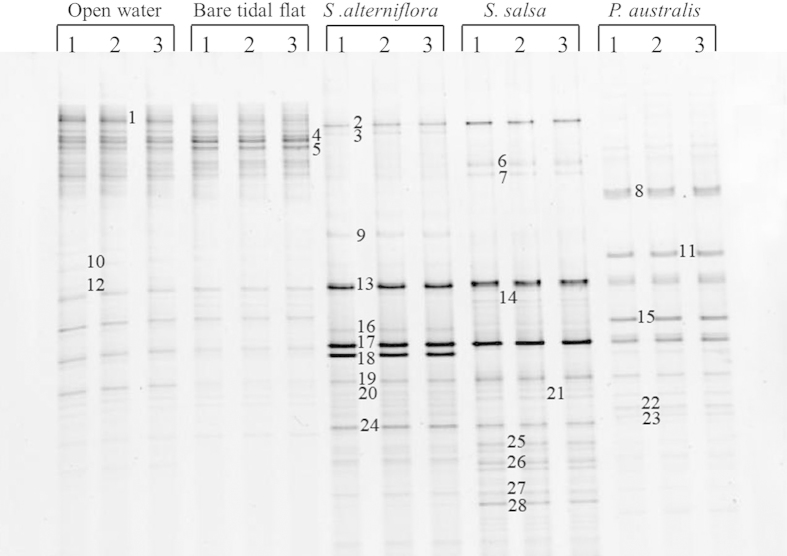
Denaturing gradient gel electrophoresis (DGGE) patterns of methanogen 16S rRNA genes amplified with the 1106F-GC/1378R primer pair from DNA extracts obtained from salt marsh samples. The denaturant gradient range is 45–75%.

In the DGGE examination of the archaea 16S rRNA genes, a total of 28 bands were observed in samples from the five transect regions (between 14 and 19 bands per region; Fig. 1). The mobility and intensity of the DGGE bands differed among regions. In the open water sediments and the bare tidal flat, the bands with strongest intensity tended to be concentrated at the top of the gel (bands 2, 4, 5 and 7 in Fig. 1). In contrast, the most intense bands migrated to the bottom of the gel in *S. alterniflora* and *S. salsa* marshes (bands 13, 17, and 18 for *S. alterniflora* marsh and bands 13 and 17 for *S. salsa* marsh; Fig. 1). Bands 8, 11, 13, 15 and 17 were dominant in *P. australis* marsh (Fig. 1). The differences in the mobility and intensity of the DGGE bands were reflected in methanogen diversity. The *H’* value showed that the unvegetated regions had higher methanogen 16S rRNA diversity than did the vegetated regions (Table 2). The lowest methanogen diversity was observed in the *S. alterniflora* marsh, though this was not significantly lower than that observed in the *S. salsa* and *P. australis* marshes (Table 2). Log-*H’* values were negatively correlated with SOC and showed a tendency to be negatively correlated with acetate concentration (*P* < 0.10), but were not correlated with DOC, trimethylamine or formate concentrations.

In total, 28 bands were sequenced, from which we identified members of *Methanomicrobiales, Methanobacteriales, Methanosaetaceae, Methanosarcinaceae* and *Halobacteriaceae* (Fig. S2). The abundance of hydrogenotrophic *Methanomicrobiales* and *Methanobacteriales* was highest in the *P. australis* marsh (Table 2). The *S. alterniflora* marsh contained the greatest abundance of acetotrophic *Methanosaetaceae*: 0.17–8.29 times higher than that of the other regions (Table 2). In the *P. australis* marsh, the open water and the bare tidal flat, however, the dominant methanogens were *Methanosaetaceae*. Methanogens belonging to *Methanosarcinaceae* were detected in all the regions with the largest abundance observed in the vegetated regions. In the *S. alterniflora* marsh, the abundance of *Methanosarcinaceae* was 2.30–56.6 times higher than in the other regions. Consequently, the *S. alterniflora* marsh had the highest relative abundance of *Methanosarcinaceae* across the transect. *Methanosarcinaceae* were also the dominant methanogens in the *S. salsa* marsh.

**Figure 2:**
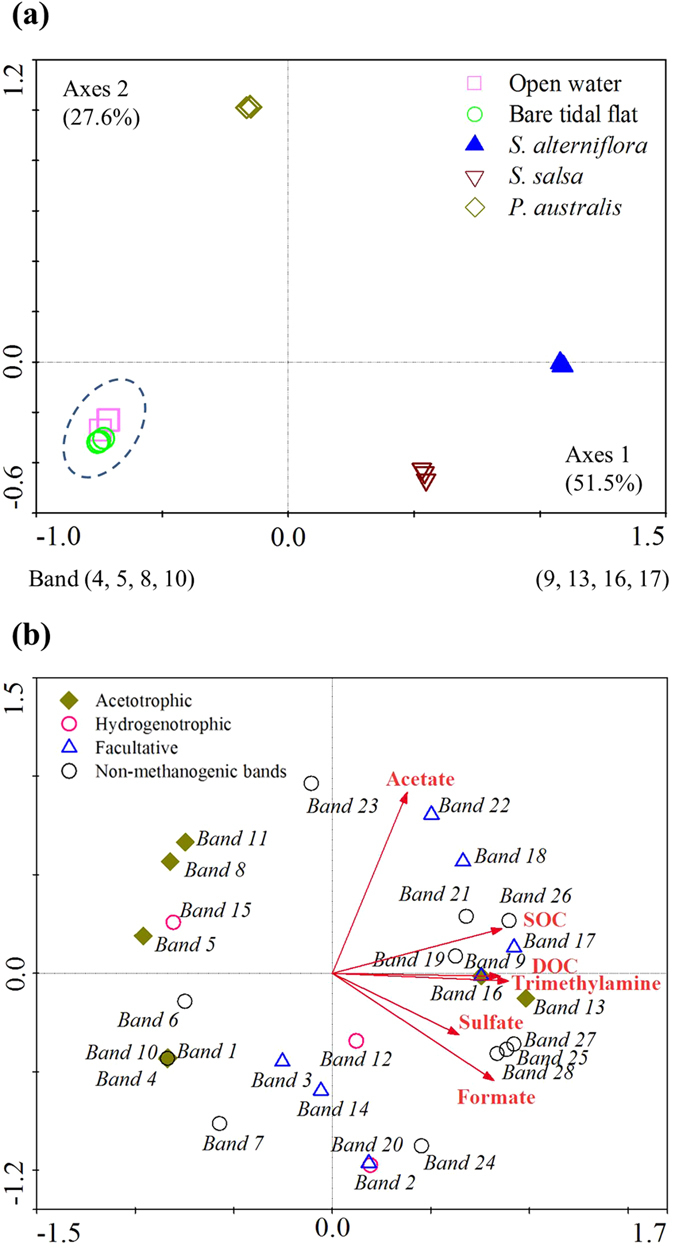
Results of (**a**) a principal component analysis and (**b**) a redundancy analysis showing ordination diagrams of the methanogen communities determined by 16S rRNA gene-DGGE. The percentage of community composition data accounted for by each axis is shown in parentheses and bands in parentheses indicate the DGGE bands that characterize differences on axis 1. Arrows indicate environmental factors and their relative effects on methanogen communities.

**Figure 3:**
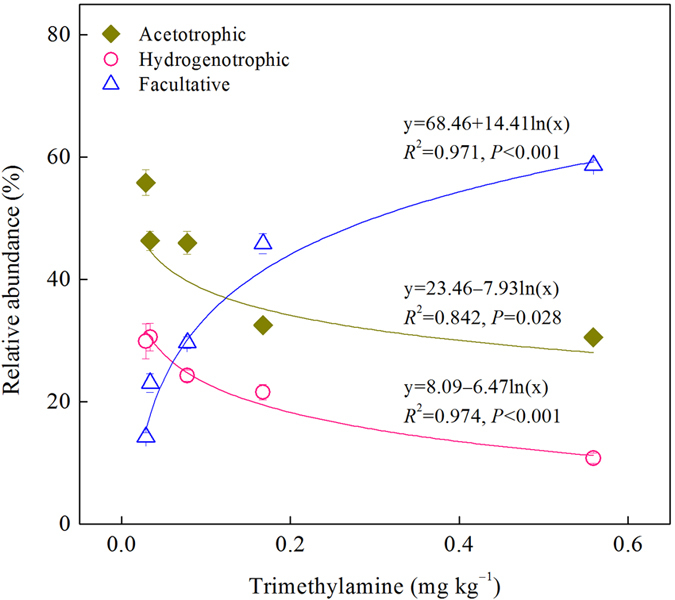
Relationship between the relative abundance of three methanogen groups and trimethylamine concentration in the coastal salt marsh. The vertical and horizontal bars denote standard errors of the means (*n* = 3).

The first ordination axes of the PCA analysis explained 51.5% of the community data (Fig. 2a). These results revealed that the two unvegetated regions had very similar methanogen communities that differed significantly from those of the vegetated regions. Methanogen communities also differed significantly amongst the three vegetated regions, primarily as result of differences in Bands 4, 5, 8 and 10 (*Methanosaetaceae*) and bands 9, 13, 16 and 17 (*Methanosaetaceae* and *Methanosarcinaceae*). The results of the RDA ordination analysis revealed that RDA axes 1 was significantly correlated with trimethylamine concentration (*R*^2^ = 0.799, *P* = 0.041, *n* = 5) and marginally correlated with SOC (*R*^2^ = 0.742, *P* = 0.061, *n* = 5) and DOC (*R*^2^ = 0.726, *P* = 0.067, *n* = 5; Fig. 2b). No significant relationship was observed between RDA axes 1 and sulfate, formate or acetate concentrations. Both the abundance and relative abundance of *Methanosarcinaceae* were significantly correlated with trimethylamine concentration (Table 3 and Fig. 3). In addition, the relative abundances of acetotrophic and hydrogenotrophic methanogens were logarithmically and negatively correlated with trimethylamine (Table 3).

### Response of CH_4_ production potential to substrates addition

**Figure 4:**
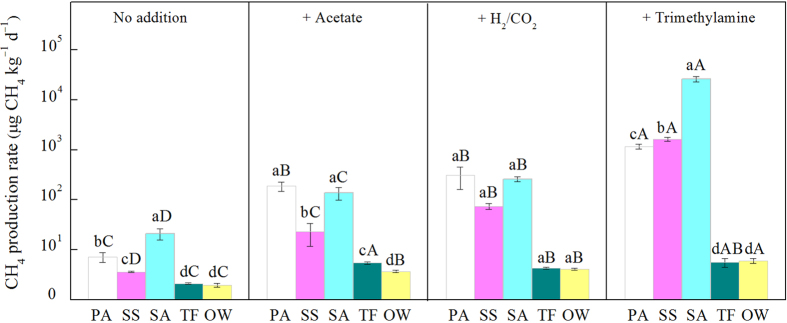
CH_4_ production rate of the coastal salt marsh as affected by the addition of acetate, H_2_/CO_2_ or trimethylamine. The vertical bars denote standard errors of means (*n* = 3). PA, *Phragmites australis*; SS, *Suaeda salsa*; SA, *Spartina alterniflora*; TF, bare tidal flat; OW, open water. Different letters denote significant differences among regions given the same additive (a, b, c, d) and among substrates within the same site (A, B, C, D).

The average of CH_4_ production potential in the salt marsh ranged from 1.95 to 20.8 μg CH_4_ kg^−1^ d^−1^, with the highest CH_4_ production potential occurring in the *S. alterniflora* marsh and the lowest in the unvegetated regions (Fig. 4). CH_4_ production potential in the *P. australis* marsh was significantly greater (7.14 μg CH_4_ kg^−1^ d^−1^) than in the *S. salsa* marsh (5.09 μg CH_4_ kg^−1^ d^−1^). When data from the five regions were combined, CH_4_ production potential was significantly correlated with SOC, DOC and trimethylamine concentrations and marginally correlated with sulfate concentration, but not with acetate or formate concentrations (Table 3). An exponential relationship was observed between CH_4_ production potential and the ratio of trimethylamine to sulfate (Table 3). Regression analysis showed that CH_4_ production potential tended to decrease with an increase in *H’* and was significantly correlated with both the abundance of methanogens and the relative abundance of facultative rather than acetotrophic or hydrogenotrophic methanogens (Table 3).

**Figure 5:**
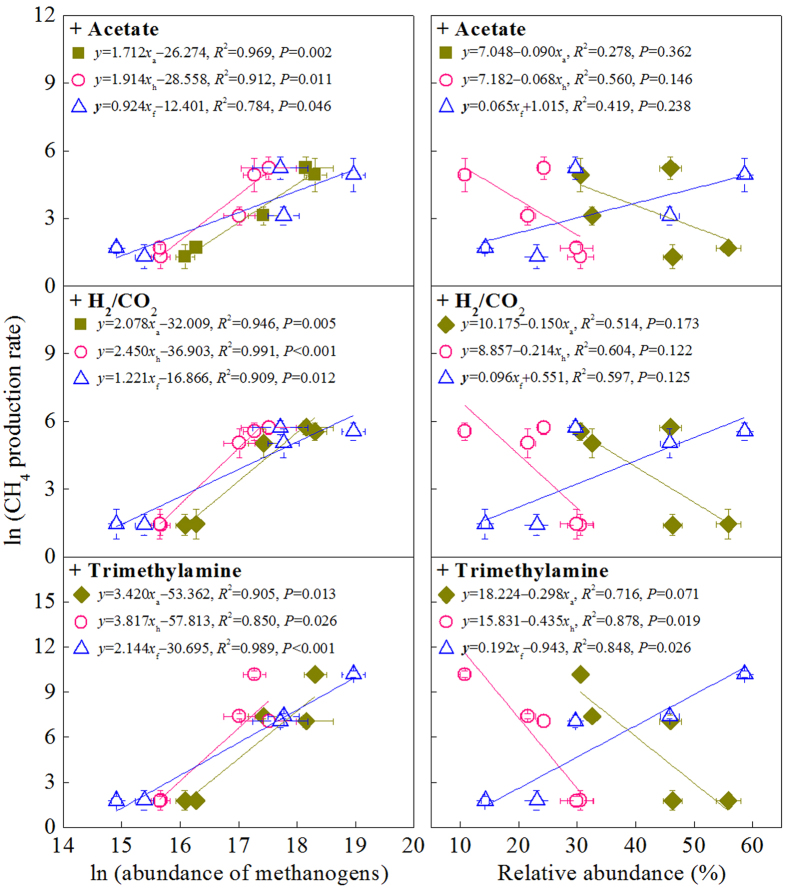
Relationships between the ln(CH_4_ production rate), *y*, and the relative abundance or ln(abundance) of acetotrophic (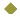, *x*_a_), hydrogenotrophic (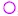, *x*_h_) and facultative (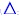, *x*_f_) methanogens under different treatments (i.e. the addition of acetate, H_2_/CO_2_ or trimethylamine). The vertical and horizontal bars denote standard errors of means (*n* = 3).

Large differences in the responses of CH_4_ production rate to substrate additions were observed between regions and between substrate types (Fig. 4). The addition of acetate, H_2_/CO_2_ and trimethylamine only slightly increased CH_4_ production in the unvegetated regions, but significantly stimulated CH_4_ production in the vegetated regions (Fig. 4). The addition of trimethylamine produced a stronger effect than did the addition of acetate and H_2_/CO_2_ in the vegetated regions. In the *S. alterniflora* marsh, the addition of acetate induced a 5.61-fold increase in methanogenesis, while the addition of H_2_/CO_2_ induced a 11.4-fold increase, and the addition of trimethylamine induced a 1255-fold increase (Fig. 4). When the data from the five regions were combined, the (log) CH_4_ production rates of the acetate, H_2_/CO_2_ and trimethylamine treatments were all significantly correlated with the (log) abundance of acetotrophic, hydrogenotrophic, and facultative methanogens (Fig. 5). The CH_4_ production rate of the trimethylamine treatment was significantly correlated with the relative abundance of facultative methanogens but negatively correlated with the relative abundance of acetotrophic and hydrogenotrophic methanogens.

## Discussion

The invasion of exotic *S. alterniflora* to the coastal marsh significantly increased CH_4_ production potential by 192–967% above that recorded in the non-invaded regions. This finding is consistent with previous *in situ* measurements at a *S. alterniflora*-invaded site showing a 57.4–505% increase in CH_4_ emissions in coastal areas of China^28^. These findings are likely due to *S. alterniflora* having a higher plant biomass and stem density than does the native *P. australis*^10^. CH_4_ production in wetland ecosystems is known to be potentially limited by the availability of organic Carbon^13^. Dominant plant species regulate net primary production, litter quantity and chemistry, and there is a direct species-dependent link between plant production and substrates for CH_4_ production^29^. Ding *et al.*^30^ found that plant species regulated the spatial variation of CH_4_ emissions in a freshwater marsh in the Sanjiang plain of China. In this study, *S. alterniflora* had significantly higher aboveground biomass than did *S. salsa* and *P. australis*, resulting in greater accumulation of SOC and DOC in the invaded marsh^6^. We found that SOC and DOC accounted for 99.6% and 98.0% of the spatial variation of CH_4_ production potential among the five regions, respectively (Table 3). Minamikawa *et al.*^31^ similarly demonstrated that differences in CH_4_ emissions among five paddy fields were due to variation in SOC concentrations. In contrast, the invasion of European *P. australis* on the east coast of the United States did not increase SOC storage, resulting in no apparent differences in CH_4_ emissions between invasive *P. australis* and native *S. alterniflora*^11^. Thus, it is likely that the *S. alterniflora* invasion provided more substrates for methanogens by accelerating SOC accumulation and subsequently increasing CH_4_ production.

We report that the 5.94 to 7.13-fold greater concentration of DOC in the *S. alterniflora* marsh than in the unvegetated regions produced 9.00–9.67 times the magnitude of CH_4_, indicating that DOC in the *S. alterniflora* marsh was more efficiently converted into CH_4_ than in the unvegetated regions. Ström *et al.*^32^ found that easily degradable organic C from fresh plant litter, root debris and exudates was the major source of methanogenic substrates in peatlands. In bare tidal flats and open water sediments, sand-combined organic C is aged and less bioavailable^33^ and contains long-chain *n*-alkanols^34^. Thus, it is feasible that the low quantity and quality of DOC likely suppressed methanogenesis in the unvegetated regions.

We found a significant relationship between CH_4_ production potential and trimethylamine (other than acetate), as well as greater CH_4_ production in response to added trimethylamine than to added H_2_/CO_2_ or acetate. These findings indicate that CH_4_ production was primarily associated with trimethylamine in the coastal salt marsh. King^35^ similarly suggested that acetate was not a significant precursor of CH_4_ unless sulfate concentrations were lower than 1 mM. This was because the rate of sulfate reduction was 100 to 1000-fold greater than methanogenesis^36^. In this study, as sulfate concentrations in the coastal salt marsh were higher than this threshold value, methanogens may not use acetate as a substrate. In contrast, ‘non-competitive’ substrates such as methanol and methylated compounds are suggested to be preferentially utilized by methanogens^19^,^37^. It has been estimated that between 61% and 90% of the CH_4_ produced in tidal salt marshes originated from trimethylamine metabolism^38^,^39^. *S. alterniflora*, like other halophytes, can osmoregulate via the synthesis of proline, glycine betaine and dimethylsulphoniopropionate^40^. *S. alterniflora* releases methylated amines, especially trimethylamine, to salt marsh soils that can act as CH_4_ precursors during senescing^21^. We observed that acetate concentration in the fall (when *S. alterniflora* begins to senesce) was similar to that recorded in early summer^41^. However, trimethylamine concentration was approximately 8-fold higher in the fall than in the summer, and accompanied by a 5.93-fold increase in CH_4_ production potential. Therefore, we argue that trimethylamine is the key substrate for methanogensis in the *S. alterniflora* marsh.

*S. alterniflora* absorbs sulfur from tidewater for growth^42^ resulting in a significant increase in soil sulfate concentration (Table 1). In this study, we found that CH_4_ production potential increased exponentially with the ratio of trimethylamine to sulfate, indicating that sulfate accumulation suppressed net CH_4_ emissions in field. Previous studies have shown that sulfate-reducing bacteria are unable to use trimethylamine^43^. Thus, we speculate that part of the CH_4_ produced was consumed by anaerobic oxidation of CH_4_ (AOM). Sulfate has been proposed to be the terminal electron acceptor of AOM in marine sediments^44^, with >90% of CH_4_ produced in oceanic sediments estimated to be consumed by sulfate-coupled AOM^45^. However, whether AOM-regulated CH_4_ emission occurs in the coastal salt marsh remains unclear, as aerobic methylotrophs are relatively unimportant in hypersaline environment^46^. Buckley *et al.*^47^ demonstrated that in the microbial mats of the Great Sippewissett Salt Marsh, AOM occurred only when CH_4_ concentrations were sufficient, suggesting a threshold ambient CH_4_ concentration is needed for AOM. Thus, it is possible that the *S. alterniflora* invasion might synchronously increase the capacity of methanogenesis and AOM, suppressing CH_4_ emissions from the invaded ecosystem. Further study is required to better understand the role of AOM in the overall CH_4_ dynamics in coastal marshes.

Despite very high methanogen abundance in the *S. alterniflora* marsh (0.84–12.6 times greater than in the other regions), methanogen diversity was low in this region. This indicates that the *S. alterniflora* invasion fundamentally altered methanogen communities. We found that the increase in the abundance of methanogens in the invaded ecosystem was asynchronous among the three methanogenic groups. Acetotrophic *Methanosaetaceae*, hydrogenotrophic *Methanomicrobiales* and *Methanobacteriales* remained at relatively similar in the invaded *S. alterniflora* marsh and the native *P. australis* or *S. salsa* marshes. However, in the *S. alterniflora* marsh, the abundance of facultative *Methanosarcinaceae* was almost 250% greater than in the *S. salsa* and *P. australis* marshes, such that *Methanosarcinaceae* became the dominant methanogens. This is in stark contrast to the unvegetated site where the acetotrophic *Methanosaetaceae* dominate^48^. Methanogen communities are suggested to be regulated by the availability of substrates^14^. Obligate, high-affinity acetotrophic *Methanosaetaceae* are described as slow-growing methanogens, while *Methanosarcinaceae* are fast-growing with a higher minimum threshold (0.2–1.2 mM) than *Methanosaetaceae* (7–70 μM) when growing on acetate^49^. Thus, *Methanosarcinaceae* generally tend to predominate over *Methanosaetaceae* in substrate-rich freshwater wetlands^12^. For example, Liu *et al.*^14^ observed that the dominant methanogens shifted from *Methanosaetaceae* to *Methanosarcinaceae* and then to *Methanobacteriales* along an increasing DOC gradient in freshwater wetlands. Peng *et al.*^50^ also reported that an increasing abundance of *Methanosaetaceae* coincided with a decrease in acetate concentrations to <200 μM in rice paddy soil. In this study, although the *S. alterniflora* invasion significantly increased acetate concentration compared with the unvegetated regions, the concentration of acetate in the *S. alterniflora* marsh remained an order of magnitude lower than the threshold value of 0.2 mM. Therefore, the change of methanogen community structure in the invaded ecosystem could not be attributed to the accumulation of acetate. The results of our regression and RDA analyses indicated that trimethylamine was the primary factor regulating methanogen community structure. *Methanosarcinaceae* are metabolically diverse but they are the only methanogen group capable of utilizing methylamines. Besides amines derived from the decomposition of *S. alterniflora*, benthic animals and phytoplankton also contain high amine concentrations and could be sources of amines to salt marshes either by direct release or during decomposition^19^,^21^. Wang and Lee^51^ demonstrated that trimethylamine consumption is the primary pathway for methanogenesis by *Methanosarcina, Methanococcus* and *Methanomethylovorans* in salt marsh and mangrove sediments.

We observed a significant association between the relative abundance of *Methanosarcinaceae* and CH_4_ production potential or CH_4_ production rate under the added trimethylamine treatment (see Fig. 5), suggesting that *Methanosarcinaceae* play a key role in CH_4_ production by utilizing trimethylamine in the salt marsh. This suggestion is further supported by our observation of a greater initial response of methanogenesis to added trimenthylamine than to added H_2_/CO_2_ or acetate in the *S. alterniflora* marsh. Purdy *et al.*^37^ similarly reported that the addition of trimethylamine induced a rapid proliferation of *Methanosarcinales* rather than *Methanomicrobiales* and a pulse of CH_4_ emission from sediments in a British estuarine ecosystem. Parkes *et al.*^19^ demonstrated that the turnover rate of trimethylamine to CH_4_ was 2–4 orders of magnitude greater than that from either acetate or bicarbonate in the *Methanosarcinales-*dominated surface sediment of British salt marshes. Interestingly, in this study, the addition of acetate and H_2_/CO_2_ did stimulate CH_4_ production in the vegetated regions even when sulfate concentration in the overlying water was 30 mM. In contrast, despite harboring the highest relative abundance of *Methanosaetaceae*, the response of CH_4_ production to the added acetate treatment in the unvegetated regions was much lower than that observed in the vegetated regions. Senior *et al.*^52^ suggested that the ‘sulfate-depleted’ microzones could be structured at vegetated sites due to a large reduction in sulfate. The presence of plant issue and debris may supply labile organic substrates and promote sulfate reduction exceeding the rate of sulfate replenishment. In this way, acetotrophic and hydrogenotrophic methanogens can survive and sustain methanogenesis activities in microzones^35^. In contrast, in the bare tidal flat and open water sediments, the lack of substrates and suitable microzones would result in the proliferation and activity of acetotrophic and hydrogenotrophic methanogens at low levels.

In summary, the invasion of exotic *S. alterniflora* to this coastal marsh ecosystem significantly increased CH_4_ production potentials by 192–968% above that observed regions with native species and in unvegetated regions. *S. alterniflora* significantly increased the supply of ‘non-competitive’ substrate trimethylamine for methanogens, which in turn stimulated the proliferation of facultative *Methanosarcinaceae* rather than acetotrophic and hydrogenotrophic methanogens. This resulted in a shift of the dominant methanogens from acetotrophic to facultative. Thus, we conclude that the increase of CH_4_ production under the *S. alterniflora* invasion was the result of an increase in the ‘non-competitive’ trimethylamine and a shift in methanogen community structure.

## Methods

### Study site

The coastal salt marsh of the Yancheng National Nature Reserve (33°22′N, 120°42′E), Yancheng city, Jiangsu Province, China (Fig. S1) has a warm temperate climate, with a mean annual air temperature of 12.6 °C and a mean annual precipitation of 1040 mm. Tides in this region are semidiurnal with an amplitude of 2–3 m and seawater salinity is 30.0–32.0%. *S. alterniflora* was intentionally introduced to this site in 1982 from the United States to control coastal erosion^22^. Since then it has rapidly spread and gradually replaced native *P. australis* and *S. salsa*, resulting in a substantial shift in plant zonation across the marsh landscape. Coastal wetlands in the area are composed of five regions: open water, tidal mudflat, *S. alterniflora, S. salsa* and *P. australis*. Open water is exposed only during extreme low tides or full moon events. The bare tidal flat and the *S. alterniflora* marsh are located on the lower and middle regions of the intertidal zone, respectively, and are inundated semidiurnally. The *S. salsa* marsh occurs in the irregularly flooded high intertidal zone and is inundated only when the tidal level is higher than the mean high water spring tide. The *P. australis* marsh is located in the rarely flooded supralittoral zone and is inundated only after the occurrence of storms and heavy rainfall events. An experimental area spanning each of the five regions was mapped with a surveyor’s transit and laser rangefinder, and a spatially planimetric map of the site was prepared using the Surfer^®^ 12^53^.

### Sampling and analyses

Plant and soil samples were collected on 22 September 2012. The *S. alterniflora* site was selected where *S. alterniflora* replaced *S. salsa* in 2002. Three 50 cm^2^ or 25 m^2^ sampling quadrats were randomly established in each region. Plants in each of the smaller quadrats were clipped at the soil surface. Plants were washed carefully with distilled water and oven-dried at 70 °C for measurement of aboveground biomass. Soil samples (0–20 cm) were collected at ten different positions in each of the larger quadrats using a stainless steel soil sampler (2.5 cm diameter). All soil samples were stored in a cool box and transported to the laboratory for analysis. Soil subsamples for DNA analysis were stored at −20 °C for DNA extraction. A subsample (~200 g) was also air dried for analysis of soil properties.

The concentrations of SOC and total N (TN) were determined via the wet oxidation redox titration method and the micro-Kjeldahl method, respectively. The dissolved organic and inorganic components of fresh soils were extracted by deionized water using a (oven-dried) soil to water ratio of 1:2 (w/v) for 30 min under agitation at 200 rpm in a flask, and then centrifuged at 4,000 rpm for 25 min at 4 °C. The supernatant was immediately acidified with 2 mL 2.0 M HCl and filtered through a 0.45-μm membrane filter (Whatman, Clifton, NJ, USA). A 100 mL subsample of the extract was frozen for amine analysis^21^. Dissolved organic carbon (DOC) in the extracts was analyzed on a Shimadzu C analyzer (TOC Vcph, Shimadzu, Kyoto, Japan). Acetate and formate were determined by high-performance liquid chromatography (LC-2010HT, Shimadzu, Kyoto, Japan) fitted with a Shodex RS-Pak KC-811 column (Waters Corporation, Milford, MA, USA) and an ultraviolet-visible spectrum detector (SPD-20A/20AV, Shimadzu, Kyoto, Japan). Sulfate (SO_4_^2−^) was analyzed using a DX-120 ion chromatography (Dionex, Camberley, UK). Methylated amines were concentrated to approximately 7–8 mL from 100 mL of acidified extracts by diffusion at 55 °C for 24 h. An aliquot (6 mL) of concentrated extract was pipetted into a 10 mL vial and supplemented with 2 g NaCl. Vials were sealed with Teflon-faced septa before injection of 0.5 mL of 10 M NaOH. The analysis of methylated amines was performed by injecting a 1.0 mL headspace sample into a gas chromatography-mass spectrometry (GC-MS) system that consisted of a helium CP 3800 GCs (Varian, Darmstadt, Germany) in combination with a Saturn 2200 MS (Varian, Darmstadt, Germany). The injector, detector, and transfer line were set at 120 °C, 250 °C, and 280 °C, respectively. The column temperature was initially held at 35 °C for 3 min, then increased at a rate of 35 °C min^−1^ to 120 °C and maintained at this temperature for 2 min to remove volatile or semi-volatile interference before the next injection. The flow rate of the helium carrier gas was set at 1 mL min^−1^ using the splitless mode. Electron impact mode at 70 eV was used, and quantification of amines was performed under the selected ion monitoring mode^41^.

### Measurement of CH_4_ production potential

The CH_4_ production potential of wetland soils was determined using the method of Galand *et al.*^54^ with slight modification. Briefly, 10 g of the fresh soil samples (oven-dried) were added to a 100 mL incubation jar and the ratio of soil to water was modified with artificial seawater to 1:2. The artificial seawater consisted of 20 g of NaCl, 4.26 g of Na_2_SO_4_, 3 g of MgCl_2_·2H_2_O, 0.2 g of CaCl_2_·2H_2_O, 0.2 g of KH_2_PO_4_ and 0.5 g of KCl per liter. The solution was buffered at pH 7.2 with 10 mM 3-(N-morpholino)propanesulfonic acid. The artificial seawater was autoclaved and deoxygenated with N_2_. Immediately prior to dispensing, NaHCO_3_ was added to a concentration of 10 mM, Na_2_S was added to a concentration of 300 mM and resazurin was added to a concentration of 1 mg l^−1 ^47. The anoxic artificial seawater was then dispensed into jars and the contents thoroughly mixed to form sludge. The jars were sealed with butyl rubber septa, evacuated with a vacuum pump, and back flushed with high purity N_2_ using an atmospheric pressure balance. The evacuation/backflush procedure was repeated three times to obtain completely anoxic conditions. After that, a stimulation experiment with four treatment conditions (no additions, H_2_/CO_2_ added by removing 22.4 mL of headspace N_2_ and injecting 22.4 mL H_2_/CO_2_ mixture (80/20%), 10 mM sodium acetate added, or 4.44 mM trimethylamine added) was conducted in the dark at 25 °C for 240 h. All four treatments were performed in triplicate. During the incubation, CH_4_ concentrations in the headspace were measured daily by sampling the headspace of the jars with a syringe. These gas samples were analyzed by gas chromatography (GC12A, Shimadzu, Kyoto, Japan). CH_4_ production potential was calculated from the slope of the linear regression given by the CH_4_ concentration increase over time.

### DNA extraction and PCR amplification

The total DNA of composite soil samples obtained from each plot was extracted with the FastDNA SPIN Kit (Bio 101, Vista, Carlsbad, CA, USA), according to the manufacturer’s instructions^55^. The 16S rRNA gene was chosen as a molecular marker to study methanogen community structure^56^. The primer pair 1106F (5′-TTW AGT CAG GCA ACG AGC-3′) and 1378R (5′-TGT GCA AGG AGC AGG GAC-3′) was used for PCR amplification. A GC clamp (5′-CGC CCG CGC GCG GCG GGC GGG GCG GGG GCA CGG GGG G-3′) was added to the forward primer to enable denaturing gradient gel electrophoresis (DGGE) analysis. The 25 μL PCR reaction mixture consisted of 12.5 μL Premix Taq version 2.0 (TaKaRa, Dalian, China), 0.25 μL of each primer (50 pmol), 1 μL of 5-fold diluted DNA template and 11 μL sterilized distilled water. The reaction was initiated by denaturing at 94 °C for 5 min, followed by 35 cycles of denaturing at 94 °C for 30 s, annealing at 55 °C for 30 s and extension at 72 °C for 90 s, with a final extension at 72 °C for 5 min.

Real-time PCR was carried out using the primer pair 1106F/1378R to quantify the abundance of methanogen 16S rRNA genes in soils using a LightCycler ST300, LightCycler Software Version 3.5 (Roche Diagnostics, Germany) and SYBR Premix Ex Taq (TaKaRa, Dalian, China). Each reaction mixture (25 μL) consisted of 12.5 μL 1 × SYBR Premix Ex Taq, 0.25 μL of each primer, 1 μL of DNA template diluted 5-fold, and sterilized distilled water. The real-time PCR program was initiated by a denaturing step at 95 °C for 10 min, followed by 35 cycles of denaturing at 95 °C for 10 s, annealing at 57 °C for 10 s, and extension at 72 °C for 6 s. A standard curve based on known methanogens 16S rRNA gene copy numbers (1.97 − 19.7 × 10^8^ copies μL^−1^) was generated using the purified PCR product.

### Denaturing gradient gel electrophoresis

DGGE was performed using a Dcode Universal Mutation Detection System (BioRad Laboratories, Hercules, CA, USA), according to the manufacturer’s instructions. PCR products (8 μL) were loaded on gels containing 8% (w/v) polyacrylamide, 1 × TAE buffer and linear gradients of 45% to 70% denaturant (100% denaturant consisted of 7 M urea and 40% (v/v) formamide). Electrophoresis was performed at 60 °C and 20 V for 10 min, followed by 70 V for 18 h. Gels were stained with SYBR Green Ι (1:10,000 dilution; Biowhittaker Molecular Applications, Rockland, ME, USA) for 30 min, rinsed with dilution TAE buffer, and photographed under UV illumination with Polaroid Type 665 positive/negative film. All samples (three replicates) for each comparison were run on the same gel. The position and intensities of individual bands on the DGGE profiles was determined using Quantity One 4.4 gel documentation software (BioRad). DGGE profiles of methanogen communities were analyzed by subtracting the background fluorescence from each lane. Band intensities were normalized to the total intensity of all bands in a given lane, resulting in relative band intensities^15^. This value indicated the relative abundance of each group under the defined PCR conditions^57^.

### Cloning, sequencing and phylogenetic analysis

The nucleotide sequences of the DNA fragments that were recovered from the bands on DGGE gels were determined as follows. First, the positions of bands were manually marked with Adobe Photoshop 7.0. Next, the bands were excised with a sterile scalpel and transferred to a 1.5 mL Eppendorf tube with 20 μL of sterilized distilled water and stored overnight at 4 °C. The eluate was then used as template for PCR re-amplification under the conditions previously described using a primer pair without a GC clamp (1106F/1378R). Re-amplified products were cloned and ligated into the pEasy-T3 cloning vector (TaKaRa), according to the manufacturer’s instructions and then sequenced with an ABI 3730 DNA Automatic Sequencer using Big Dye-terminator cycle sequencing chemistry (Applied Biosystems, CA, USA). Phylogenetic relationships among the obtained sequences were determined using the BLAST search program on the NCBI web site (www.ncbi.nlm.nih.gov). This information was used to construct neighbor-joining trees, using Poisson correction distances, pairwise deletion of gaps and missing data. Bootstrapping (500 replicate reconstructions) was used to estimate the reliability of the tree reconstructions, using MEGA 4.0^58^. The obtained 16S rRNA gene nucleotide sequences have been deposited in the GenBank database (accession numbers: KP420447 - KP420474).

### Diversity of methanogens and statistical analysis

The Shannon-Weaver diversity index (*H’*) was used to determine the variation of methanogen diversity across the marsh transect:


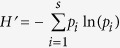


where 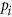 is the proportion of the *i*th band intensity in all bands in the same lane and *s* is the total number of bands. The following community ordination analyses were also conducted using relative band intensity data: a principal component analysis (PCA) and redundancy analysis (RDA) conducted using Canoco for Windows 4.5 (Microcomputer Power, Ithaca, NY, USA). The dissimilarity of the methanogen communities by PCA among different regions was tested with Wilcoxon signed-rank test using the first principal component scores^59^. For RDA ordinations, biplot scaling was used by focusing on inter-sample distances. Environmental variables considered in these ordination analyses included SOC, TN, DOC, acetate, formate, sulfate and trimethylamine and plant aboveground biomass. A stepwise forward selection method was used to choose variables for inclusion in the final model and a Monte Carlo test with unrestricted permutations was used to determine the significance of the environmental variables in accounting for community structure data^60^.

We divided the 16S rRNA genes affiliated to methanogens into three groups based on sequencing results and the metabolic pathways for methanogenesis: hydrogenotrophic, acetotrophic and facultative. The proportion of each group of the total abundance of methanogens was calculated according to the relative band intensities. Statistical analyses were conducted using SPSS 11.0 (SPSS Inc., Chicago IL, USA) unless otherwise stated. All data were expressed on the basis of oven-dried soil and were evaluated for normality. If necessary, values were log-transformed prior to statistical analyses. Statistically significant differences in soil and vegetation properties and methanogen community structure characteristics amongst regions were assessed by a one-way analysis of variance (ANOVA). Regression analyses were used to test the significance of statistical associations between methanogenic substrates and SOC, and between CH_4_ production potential and soil properties or methanogen community characteristics. Spearman’s non-parametric rank correlation was used to describe the associations between the abundance of methanogens and the various soil characteristics. The fit of the linear equations was evaluated by the coefficient of determination, *R*^2^, and its statistical significance was determined by Fisher’s *F* test. The significance of the regression coefficients was tested by Student’s *t*-test.

## Additional Information

**How to cite this article**: Yuan, J. *et al.* Shifts in methanogen community structure and function across a coastal marsh transect: effects of exotic *Spartina alterniflora* invasion. *Sci. Rep.* **6**, 18777; doi: 10.1038/srep18777 (2016).

## Supplementary Material

Supplementary Information

## Figures and Tables

**Table 1:** Soil and vegetation properties in samples obtained from coastal salt marsh.

Site	Aboveground biomass	SOC	DOC	Salinity	Sulfate	Acetate	Formate	Trimethylamine
	(g m^−2^)	(g C kg^−1^)	(g C kg^−1^)	(‰)	(g SO_4_^2−^kg^−1^)	(mg kg^−1^)	(mg kg^−1^)	(mg kg^−1^)
*P. australis*	0.79 ± 0.18b	5.42 ± 0.50b	0.23 ± 0.01bc	3.16 ± 0.64d	0.25 ± 0.09c	1.91 ± 0.51a	0.37 ± 0.20a	0.08 ± 0.02c
*S. salsa*	0.41 ± 0.05c	3.95 ± 0.25c	0.32 ± 0.02b	7.75 ± 0.94c	0.47 ± 0.07bc	0.67 ± 0.11bc	0.66 ± 0.12a	0.17 ± 0.02b
*S. alterniflora*	1.70 ± 0.11a	13.55 ± 0.42a	1.07 ± 0.12a	16.06 ± 0.67a	1.22 ± 0.25a	1.17 ± 0.51b	0.65 ± 0.14a	0.56 ± 0.15a
Bare tidal flat	—	1.88 ± 0.33d	0.15 ± 0.04c	9.96 ± 0.33c	0.51 ± 0.05bc	0.39 ± 0.12c	0.47 ± 0.08a	0.03 ± 0.00d
Open water	—	1.33 ± 0.17d	0.18 ± 0.02bc	13.49 ± 1.43b	0.57 ± 0.02b	0.33 ± 0.08c	0.41 ± 0.23a	0.03 ± 0.00d
*F* value	84.7	586.4	143.8	97.4	26.4	20.5	2.3	152.4
*P* value	<0.001	<0.001	<0.001	<0.001	<0.001	<0.001	0.13	<0.001

Values are means (*n* = 3) ± standard errors. Different letters within the same column indicate significant differences at *P* < 0.05.

SOC, soil organic carbon; TN, total nitrogen; DOC, dissolved organic carbon.

**Table 2:** Abundance, relative abundance and the Shannon-Weaver diversity of methanogens in the coastal salt marsh.

Site	Abundance of methanogens ( × 10^8^ copies g^−1^ d.w.s)				Relative abundance of methanogens (%)			Shannon-Weaver
	Total	Acetotrophic	Hydrogenotrophic	Facultative	Acetotrophic	Hydrogenotrophic	Facultative	diversity (*H’*)
*P. australis*	1.94 ± 0.71b	0.77 ± 0.28a	0.40 ± 0.15a	0.49 ± 0.18b	45.99 ± 1.87b	24.31 ± 0.94b	29.70 ± 0.94c	2.21 ± 0.02c
*S. salsa*	1.73 ± 0.50b	0.37 ± 0.11b	0.25 ± 0.07a	0.52 ± 0.15b	32.56 ± 0.51c	21.58 ± 1.27b	45.86 ± 1.64b	2.20 ± 0.05c
*S. alterniflora*	3.56 ± 1.71a	0.90 ± 0.18a	0.32 ± 0.06a	1.72 ± 0.35a	30.54 ± 0.68c	10.79 ± 0.85c	58.68 ± 0.80a	2.18 ± 0.02c
Bare tidal flat	0.26 ± 0.02c	0.11 ± 0.01c	0.06 ± 0.01b	0.03 ± 0.00c	55.85 ± 2.11a	29.90 ± 2.84a	14.26 ± 0.73e	2.46 ± 0.02b
Open water	0.29 ± 0.04c	0.09 ± 0.01c	0.06 ± 0.01b	0.05 ± 0.01c	46.33 ± 1.57b	30.58 ± 2.27a	23.09 ± 1.54d	2.63 ± 0.01a
*F* value	76.5	57.2	42.6	162.1	150.5	58.7	671.8	169.2
*P* value	<0.001	<0.001	<0.001	<0.001	<0.001	<0.001	<0.001	<0.001

Values are means (*n* = 4) ± standard errors. Different letters within the same column indicate significant differences at *P* < 0.05.

The abundance of the three methanogen groups was calculated from abundance of total methanogens and the relative abundance of each group. The proportion of each group accounting for the abundance of methanogens was calculated according to the relative band intensities.

**Table 3:** Results of regression analysis to assess the significance of associations between CH_4_ production potential (y) and soil or methanogen community characteristics (x).

Soil characteristics	Equation	*R*^2^ value	*F* value	*P* value
SOC	y = 1.578x – 0.832	0.996	738.97	<0.001
DOC	y = 19.774x – 2.089	0.980	146.42	0.001
Sulfate	y = 17.669x – 3.258	0.681	6.40	0.086
Acetate	—	0.222	0.86	0.423
Formate	—	0.301	1.29	0.338
Trimethylamine	y = 34.004x + 1.516	0.942	48.93	0.006
Trimethylamine/sulfate ( × 10^3^)	y = 1.410e^5.104x^	0.892	24.84	0.016
*H’* index	y = 71792e^**−**4.098x^	0.711	7.38	0.073
Abundance of methanogens ( × 10^8^)	y = 1.643e^0.712x^	0.994	504.19	<0.001
Relative abundance of hydrogenotrophic methanogen	y = −17.990ln(x) – 19.809	0.959	69.59	0.004
Relative abundance of acetotrophic methanogens	—	0.575	4.05	0.138
Relative abundance of facultative methanogens	y = 0.955e^4.814x^	0.775	10.34	0.049
