# Supplementary material for: Shifts in methanogen community structure and function across a coastal marsh transect: effects of exotic Spartina alterniflora invasion
Source: Sci Rep. 2016 Jan 5;6:18777. doi: 10.1038/srep18777 (PMC4700438; doi:10.1038/srep18777)
Supplement: Supplementary Information [file srep18777-s1.pdf]

Shifts in methanogen community structure and function across a coastal marsh transect: effects of exotic *Spartina alterniflora* invasion

Junji Yuan[1,2], Weixin Ding[1], Deyan Liu[1], Hojeong Kang[3], Jian Xiang[1,2], Yongxin Lin[1,2]

[1] *State Key Laboratory of Soil and Sustainable Agriculture, Institute of Soil Science, Chinese Academy of Sciences, Nanjing 210008, China*

[2] *University of Chinese Academy of Sciences, Beijing 10049, China*

[3] *School of Civil and Environmental Engineering, Yonsei University, Seoul 120–749, Korea*

Author for correspondence: Weixin Ding

Tel: 0086-25-8688-1527

Fax: 0086-25-8688-1000

Email: wxding@issas.ac.cn

Supplementary information

Table S1 Correlations (Pearsons R^2 values) between the abundance of methanogens and soil or plant characteristics in the coastal salt marsh.

Abundance of methanogens	Aboveground biomass[a]	SOC	TN	DOC	Salinity	Sulfate	Acetate	Formate	Trimethylamine
Total	0.982	0.957*	0.974**	0.885*	0.174	0.628	0.628	0.583	0.903*
Acetotrophic	0.870	0.878*	0.913*	0.728	–0.067	0.411	0.855	0.276	0.732
Hydrogenotrophic	0.228	0.645	0.707	0.445	–0.416	0.056	0.935*	0.162	0.471
Facultative	0.952	0.991***	0.986**	0.977**	0.420	0.811	0.449	0.637	0.982**

* $P < 0.05$, ** $P < 0.01$ and *** $P < 0.001$.

[a] Data in the unvegetated sites were not included for analysis ($n = 3$).

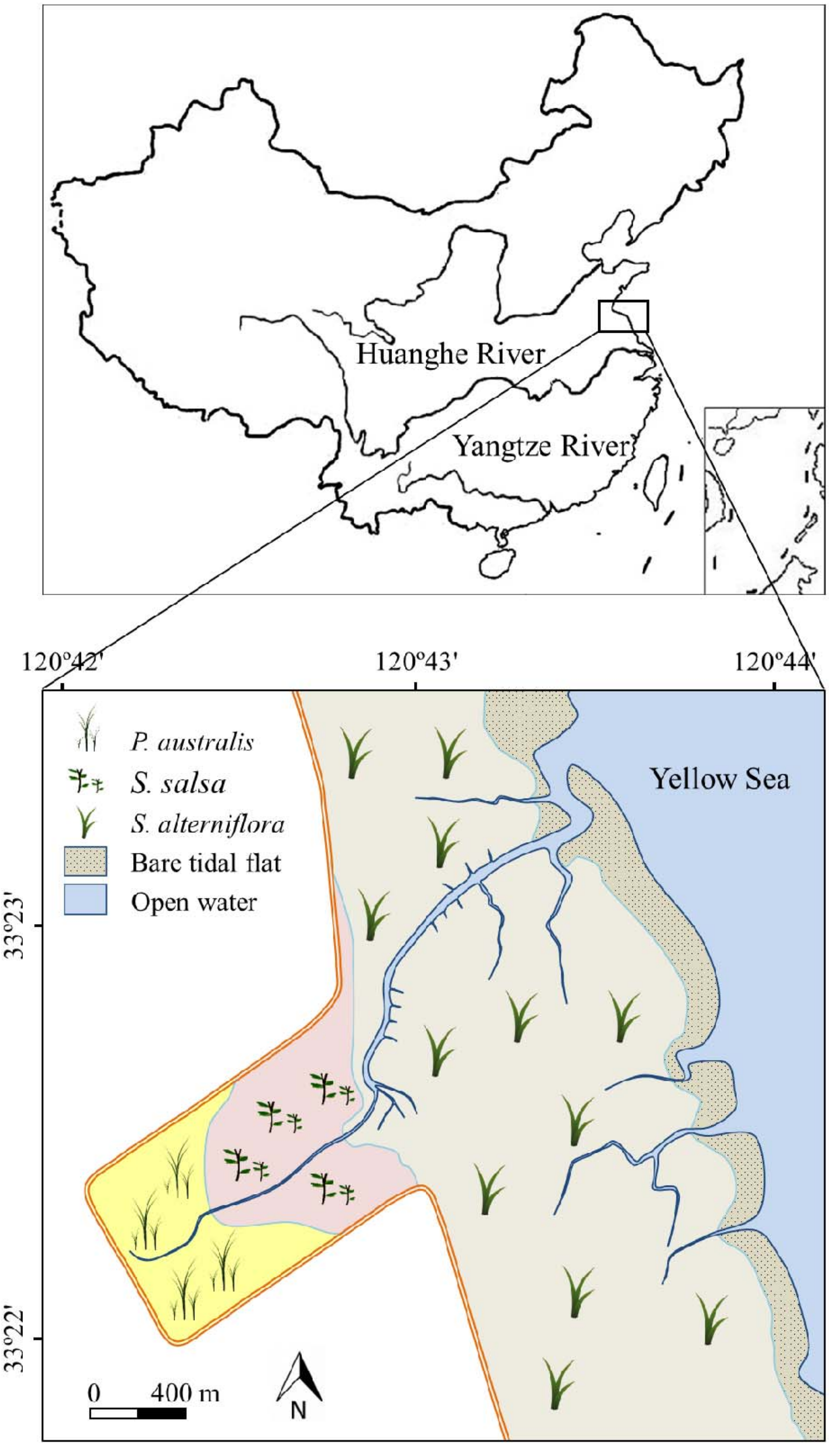

Fig. S1 Location of the sampling site in the coastal salt marsh in Jiangsu Province, China. The site map was prepared using the Surfer® 12 (Golden Software, http://www.goldensoftware.com/products/surfer).

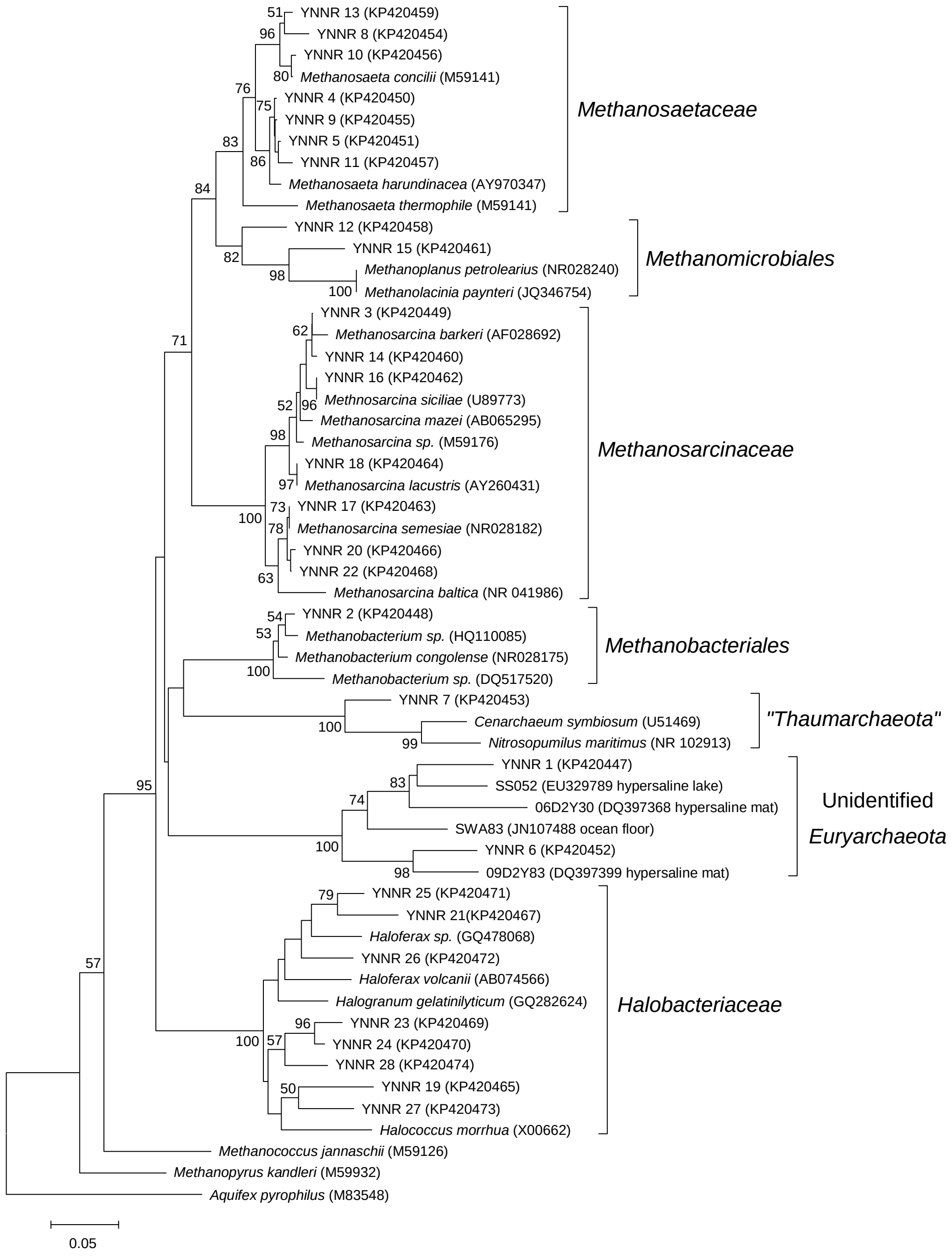

Fig. S2 Phylogenetic relationships of 16S rRNA gene sequences retrieved by DGGE with 1106F-GC/1378R (*Escherichia coli* positions 1,107 – 1,377) from the coastal salt marsh soils, using *Aquifex pyrophilus* as the outgroup. Bootstrap values >50% are shown at branch points. The scale bar represents 0.5 substitutions per nucleotide. The GenBank accession numbers of the reference sequences are indicated in parentheses.
